# On-chip ultrasensitive and rapid hydrogen sensing based on plasmon-induced hot electron–molecule interaction

**DOI:** 10.1038/s41377-023-01123-4

**Published:** 2023-03-21

**Authors:** Long Wen, Zhiwei Sun, Qilin Zheng, Xianghong Nan, Zaizhu Lou, Zhong Liu, David R. S. Cumming, Baojun Li, Qin Chen

**Affiliations:** 1 Guangdong Provincial Key Laboratory of Nanophotonic Manipulation, Institute of Nanophotonics, Jinan University, 511443 Guangzhou, China; 2 College of Life Science and Technology, Jinan University, 510632 Guangzhou, China; 3 School of Engineering, University of Glasgow, Glasgow, G12 8QQ UK

**Keywords:** Imaging and sensing, Nanophotonics and plasmonics

## Abstract

Hydrogen energy is a zero-carbon replacement for fossil fuels. However, hydrogen is highly flammable and explosive hence timely sensitive leak detection is crucial. Existing optical sensing techniques rely on complex instruments, while electrical sensing techniques usually operate at high temperatures and biasing condition. In this paper an on-chip plasmonic–catalytic hydrogen sensing concept with a concentration detection limit down to 1 ppm is presented that is based on a metal–insulator–semiconductor (MIS) nanojunction operating at room temperature and zero bias. The sensing signal of the device was enhanced by three orders of magnitude at a one-order of magnitude higher response speed compared to alternative non-plasmonic devices. The excellent performance is attributed to the hydrogen induced interfacial dipole charge layer and the associated plasmonic hot electron modulated photoelectric response. Excellent agreements were achieved between experiment and theoretical calculations based on a quantum tunneling model. Such an on-chip combination of plasmonic optics, photoelectric detection and photocatalysis offers promising strategies for next-generation optical gas sensors that require high sensitivity, low time delay, low cost, high portability and flexibility.

## Abstract (web summary)

Interface dipole modulated hot-electron ejection enables an on-chip electric readout plasmonic hydrogen sensor with abnormal S-shape *I*–*V* and advanced sensing performance at a limit of detection of 1 ppm.

## Graphical abstract

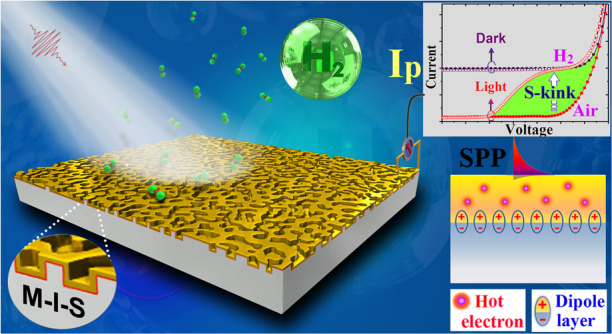

## Introduction

Hydrogen has been regarded as one of the promising next-generation energy sources to replace the fossil fuels due to its zero-carbon property and high heat of combustion (142 kJ g^−1^)^1^. Liquid hydrogen has already been used for rocket fuels and hydrogen fed vehicles. Moreover, hydrogen acting as a strong reducing agent has also been widely used in chemical industry, metal smelting, nuclear reactors, semiconductor processing and biomedical^2–5^. However, colorless, odorless and tasteless hydrogen has low minimum ignition energy (0.017 mJ), wide flammable range (4–75% in air), and low density (0.0899 kg m^−3^), which make it easy to diffuse (0.61 cm^2^ s^−1^ in air), difficult to be noticed and thus dangerous. For example, a hydrogen explosion contributed to the Fukushima accident in 2011. Therefore, accurate and rapid detection is necessary during the production, storage, transportation and use of hydrogen^6–8^. Generally, selective hydrogen sensors are based on the catalytic reactivity and solubility of hydrogen with some noble metal elements, such as palladium and platinum^9–12^. Interaction of hydrogen with the sensing element causes changes in resistance^13,14^, work function^15^, volume^8,11^, temperature^16^, and refractive index^17^, which can be used to detect and quantify the hydrogen gas concentration. Existing electrical sensing techniques such as thermal conductivity based^18^, and resistance based^13,14^, electrochemical^19^ and semiconductor junction^20^ based usually operate at high temperatures and biasing condition, which increase the system complexity, the risk of explosion and the power consumption. Optical sensing techniques including optical fiber sensing^21^ and tunable diode laser absorption spectroscopy (TDLAS)^22^ rely on complex and expensive instruments like spectrometer, tunable laser, and bulky gas cell, which needs skilled operators and complicated data demultiplexing processes. The emergence of a hydrogen economy^2^ provides the impetus to produce 3A criteria for hydrogen sensors, i.e., **A**ccessible (high integration, easy to use), **A**ffordable (low manufacturing and use cost), and **A**pplicable (high accuracy, rapid).

Plasmonic sensing is an emerging technique with promising potential to meet the 3A criteria^23–28^. Due to its strongly localized electromagnetic field at the metal surface, the interaction of surface plasmon resonance (SPR) and analytes results in significant influence of the resonance wavelength, amplitude, phase and polarization, indicating ultrahigh sensitivity^29–33^. Moreover, low-cost nanopatterning techniques such as nanoimprint^34^, nanosphere lithography^35^, and chemical synthesis^36^ have been widely used to fabricate various plasmonic sensing structures. However, plasmonic sensors rely heavily on the complex and expensive instruments such as spectrometers^25–31^ and BioCore sensing system^37^ due to the lack of in situ photoelectric conversion, although the sensor chip itself is compact. This is a common issue of optical sensors for on-site and portable applications^38,39^. Recently, an on-chip direct electric read out plasmonic sensor was proposed and demonstrated promising potential as a portable sensing platform^40^, where plasmonic metal–semiconductor nanojunction provides dual functions including plasmonic sensing and plasmon-enhanced hot electron photodetection. By interfacing metal nanostructures with carrier-accepting materials (e.g., semiconductors or molecules), the non-equilibrium carriers will be able to directly emit/tunnel through the Schottky/metal–insulator–metal barrier to form a detectable photocurrent, or transfer their energy to reactants/adsorbates for the enhanced photochemical reactions *via* reducing the activation energy or catalyzing the reaction^41–45^. However, this technique cannot be directly used for gas sensing because of the weak interaction between gas molecules and SPR^46^.

To solve this problem, in this paper a novel hydrogen sensing platform consisting of platinum–silicon nanojunctions was proposed with triple functions including plasmonic sensing, photoelectric detection and photocatalysis. Benefiting from this multifunctional combination, a unique plasmon-induced hot electron–hydrogen molecule strong interaction was observed, contributing to an abnormal S-shaped photoelectric current–voltage characteristic. And thus on-chip hydrogen detection with much higher sensitivity (three orders of magnitude) and response speed (one order of magnitude) compared to non-plasmonic MIS sensors was demonstrated at room temperature and zero bias. A quantum tunneling model considering a formation of interfacial dipole layer as the tunneling barrier for hot electron ejection was developed and the calculated photocurrent variation between different ambient gases agree well with the experimental results. It is expected that such a plasmonic–catalytic MIS nanojunction concept could provide a 3A-standard gas sensing platform with high sensitivity, high integration and low cost for portable and on-site inspection.

## Results

### Plasmonic–catalytic MIS junction

**Fig. 1:**
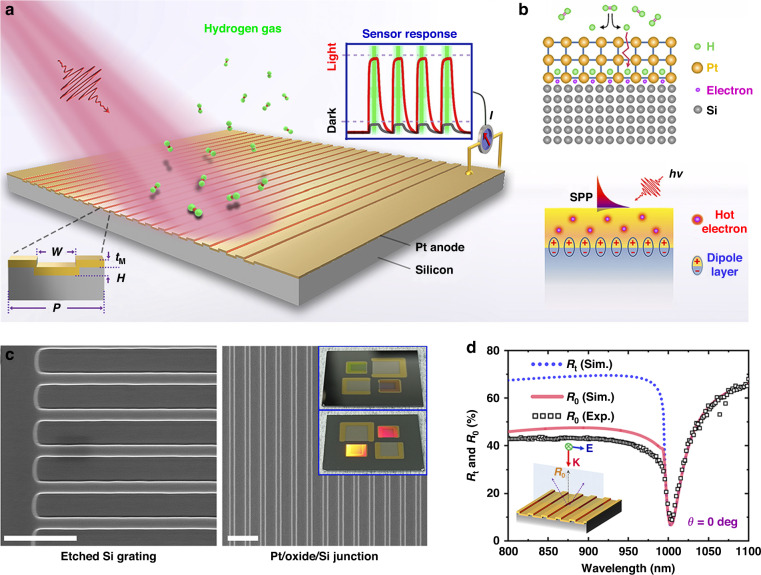
The plasmonic–catalytic MIS junction used to unravel the interplay between hydrogen molecular and hot electrons. **a** The surface-relief grating structure on silicon is coated with a conforming thin Pt layer to form the plasmonic absorber. The grating geometry of the absorber is defined by the period (*P*), width (*W*), grating depth (*H*), and the thickness of Pt (*t*_M_). **b** The adsorption and dissociation kinetics of the hydrogen molecular on the catalytic metal surface (top); Hot electron generation from plasmon decay and the electrical dipole layer formation due to hydrogen adsorption at metal–insulator interface. **c** SEM images of the grating structures before (left) and after metal deposition. The insert shows photographs of the grating sample from two different angles. **d** The experimental and numerically fitted zero-order reflection curves (*R*_0_) at normal incidence. The geometry parameters are summarized as: *P* = 1000 nm, *W* = 620 nm, *H* = 70 nm and *t*_M_ = 56 nm. These values are in good consistence with our target parameters of fabrication process or the measurements from the SEM images. The total reflectance (*R*_t_) based on the above fitting parameters are also presented in the plots

The proposed plasmonic–catalytic MIS junction is shown in Fig. 1a, where the plasmonic resonance triggers the generation of highly energetic charge carriers with a chance to be ejected into a semiconductor and form photoelectric response. Upon exposure to hydrogen gas, the adsorbed molecules at the catalytic metal surface can dissociate to hydrogen atoms more efficiently with the assistance of plasmon–molecule interaction. The separate hydrogen atoms are initially adsorbed in the interstitial lattice sites of Pt surface and then diffuse through the thin film by jumping from one interstitial site to another as driven by the concentration gradients. Since the inner metal-oxide interface is resemble to a diffusion barrier layer, further permeation is inhibited. Thus, a considerable amount of hydrogen atoms tend to accumulate at the inner surface of metal. According to previous studies^47–49^, hydrogen atoms adsorbed at the metal–oxide interface are polarized, rendering a dipole layer to be established therein via electrostatic induction as shown in Fig. 1b. The interfacial electrical properties of the MIS junction including the band alignment and the built-in potential can be significantly altered due to the presence of the hydrogen-induced dipole charges. Regarding the fact of that the interfacial electrical properties are of central importance in determining the hot carrier ejection process, the interaction of hydrogen with the plasmonic–catalytic MIS junction will eventually be reflected in the tailored photoelectric response. Gaining a microscopic insight into these processes and their interplay in such a complex plasmonic–catalytic system could aid in exploring undiscovered phenomenon and more exciting possibilities in utilization of hot carriers.

Briefly, the MIS junction is composed of a thin platinum layer (50 nm) on top of the *n*-type (1–10 Ω‧cm) silicon substrate containing one-dimensional periodic surface relief structure, which serves as the emitter and acceptor for plasmonic hot electron separation^40^. The reason to choose Pt is on the ground that this metal possesses high catalytic activity and stability allowing hydrogen molecules to adsorb and react on the surface. While the high work function Pt acts as the Schottky anode contact to n-type silicon, the rear Al-Si contact is considered as the low-resistive Ohmic cathode. As a majority-carrier device, the configuration of Schottky diodes can collect either hot electrons or hot holes, but not both. In our case, the energy band alignment of Pt and n-Si only allows for efficient ejection of hot electrons into the conduction band of the semiconductor, but leaves a very large barrier height for hot holes. Therefore, for our *n*-type silicon based device, we believe that the hot holes are unlikely to play a major role in the interface charge transfer processes. It is important to note that instead of using the metal–semiconductor (MS) configuration, MIS junction with an atomic thick intermediate oxide is considered herein for the sake of leakage current suppression and more sensitive interfacial properties. Figure 1c shows the SEM morphologies of the silicon-based surface relief gratings before and after the thin film Pt deposition. As depicted in the insets, with a proper viewing angle, vivid colors can be clearly observed in the grating areas because of its angle-dependent optical resonance properties. According to our previous studies, optical resonance behavior of such a periodically corrugated metallic surface structure can be classified to the diffuse anomaly which is more likely due to the excitation of the propagating surface plasmon mode (see Supplementary Note 1). The optical measurement was performed on the zero-order reflectance of the plasmonic structures under normal incidence as shown in Fig. 1d, which agrees well with the numerically fitted zero-order reflection curves (*R*_0_). Under polarized normal incident illumination, high contrast plasmonic resonance with peak absorption approaching 90% is revealed from the simulated total reflection (*R*_t_). In the following sections, the hydrogen-tailored optoelectric response of the plasmonic MIS junction was investigated at a fixed resonance wavelength of 1064 nm with a slight titled incident angle (3 degrees, see Supplementary Note 1 and Fig. S1 for more details).

### Hydrogen-induced abnormal photoelectric behaviors

**Fig. 2:**
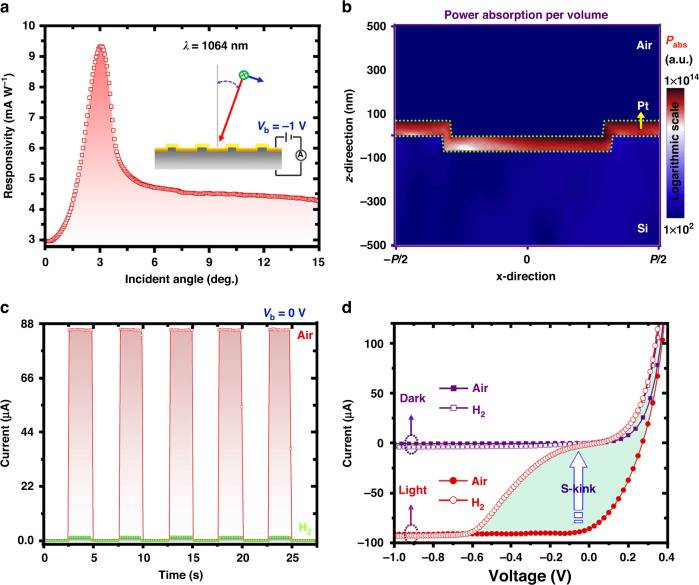
Hot electron based photoelectric conversion and the hydrogen molecular tailored response. **a** Angle-dependent photocurrent responsivity of MIS junction with a negative voltage bias (*V*_b_ = −1 V). **b** Normalized power absorption per unit volume (*P*_abs_, m^−3^) of the plasmonic structure. **c** The on-off photocurrent response of the MIS junction (*V*_b_ = 0 V) operating under air and H_2_ gas states. **d** The light and dark *I*–*V* curves of the plasmonic MIS junction after long-term exposure to air and H_2_

Upon excitation of plasmonic resonances, a series of hot carrier dynamics including generation, relaxation, transport take place in the thin-film Pt. Within the inelastic mean free path (tens of nanometer scale for most plasmonic metals), a substantial amount of generated hot carriers can reach the junction areas and then transfer into the carrier-accepting material that has low energy level. For the Schottky-type MIS contact, possible interfacial carrier separation mechanisms include thermal emission and quantum tunneling over the barrier, which result in constant photocurrent. In Fig. 2a, clear resonant characteristic was observed in the measured angle-dependent photocurrent response, which can be safely attributed to plasmonic induced light absorption as manifested by Fig. S2. The peak photocurrent responsivity (*R*_ph_) is found to be 9.4 mA W^−1^ that corresponds to an external quantum efficiency (*η*_EQE_) of 1.1%. This plasmonic-induced photoelectric response mainly arises from the collection of hot electrons generated in Pt rather than the photovoltaic conversion occurring in the underlying Si substrate, which has an EQE only around 0.02% (that corresponds to a photocurrent responsivity of 0.19 mA W^−1^, see Supplementary Note 2 for more details). As shown in Fig. 2b, optical absorption at the resonant wavelength is highly localized in the lossy metal film, resulting in significantly higher power absorption per unit volume (*P*_abs_) as compared to that of substrate.

Since the carrier dynamics involved in the interfacial hot electron transfer is ultrafast, such a photoelectric conversion scheme fundamentally exhibits a very high response speed that is solely limited by the RC parasitics of the electric structures. Experimental setup for the optoelectrical measurement is presented in Fig. S3. Figure 2c depicts the time-dependent photoelectric responses measured in air and hydrogen/air mixture (3% H_2_ in air) environments, respectively. The abrupt rise and fall in the current response due to light-on/off conditions validate the plasmonic MIS junction for fast photoelectric conversion in both gas states. Interestingly, there exists significant difference in the current on/off (light-to-dark) ratios between the results obtained in the air and H_2_-containing gas mixture environments. Apparently, a negative influence of hydrogen molecule on the interfacial carrier separation can be inferred from the considerably suppressed photocurrent response for the MIS junction exposure to the H_2_-containing mixture gas. The light and dark current–voltage (*I*–*V*) characteristics of the MIS junction in the above two gas states are presented in Fig. 2d. With the built-in potential of the MIS junction, the dark *I*–*V* curves are highly asymmetric that pointing to a clear rectifying behavior. Only a very small divergence can be observed in the two dark *I*–*V* curves. Upon light illumination, the *I*–*V* curve of the air state shifts downward into the fourth quadrant, which is identical to that of the typical junction-based photodetectors. In contrast, for the H_2_ inlet scenario, one can observe an abnormal S-shaped light *I*–*V* curve, in which a large photocurrent kink appears at small biasing voltages. The existence of the kink effect enabled by plasmon excitation can yield significantly larger changes than the dark operation conditions. To this end, one can envisage using the plasmonic–catalytic Schottky junction system as an efficient photoelectric sensing paradigm that is completely different from the conventional electrical gas sensor based on the similar combination of catalytic metals with semiconductor structure.

### Mechanisms of hydrogen-tailored interfacial hot electron transfer

**Fig. 3:**
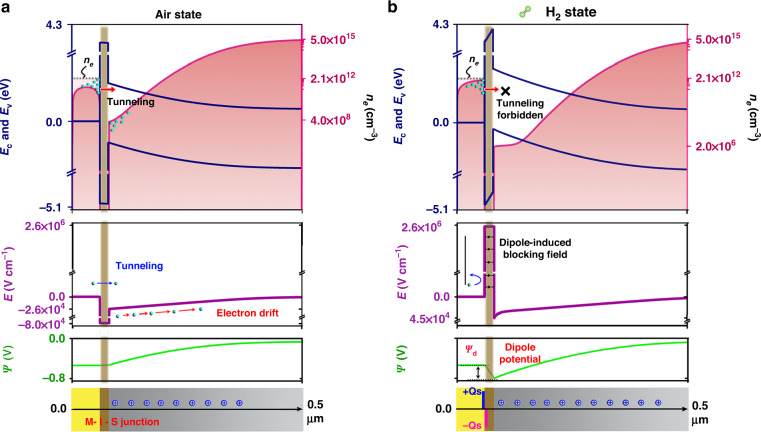
TCAD model predicted electrical characteristics of the MIS junction under different gas atmospheres. **a** In air state. **b** In hydrogen-containing gas state. The panels from top to bottom represents: energy band diagram along with the electron profiles across the MIS junctions (first row), distributions of electric field (second row) and potential (third row), and schematic of the space and dipole charge distribution near the MIS junction

Previous studies have suggested that the formation of electrical dipole layer is central to the mechanism of achieving hydrogen sensitivity in MS or MIS Schottky junction systems, as it can heavily influence the interfacial electrical properties^48,49^. Regarding this fact, it is believed that the observed abnormal *I*–*V* characteristics of the plasmonic–catalytic MIS system can be closely linked to the hydrogen-induced dipole layer. A theoretical quantum tunneling model was established for predicting the effect of a dipole charge layer on the photoelectric properties. More detailed information about the models and key parameters used in simulations can be found in Table S1. Figure 3a shows the calculated electrical characteristics of the MIS junction under inert air atmosphere. This scenario is identical to a classic MIS Schottky junction, in which the energy band alignment is mainly determined by the difference between the metal work function and the Fermi level of the *n*-type Silicon. At the silicon surface area, electrons are withdrawn from the junction (depletion condition), leaving a space charge region and a large positive built-in potential in silicon. The potential dropped on the oxide layer can be neglected regarding its ultrathin thickness. Under illumination, the initial photoexcited excess electron in the absorber has a carrier density given as *n*_e_ = *G*_opt_*τ* (*G*_opt_ is the optical generation and *τ* is the lifetime of electron), as marked with the dash line in the top plot of Fig. 3a. To reach an equilibrium state, these electrons eject from absorber to silicon by tunneling through the ultrathin oxide, as manifested by the lowered population of equilibrium electrons adjacent to the metal-oxide interface. Accordingly, with assistant by the built-in field, most of the injected electrons in silicon space charge area are immediately swept into the base region and give rise to the photocurrent. For the H_2_ case shown in Fig. 3b, an additional dipole layer is added to the metal-oxide interface by assigning exactly the same numbers of positive and negative charges for both sides. A reverse dipole potential is present in the thin oxide and impedes the flow of electrons at zero or low biases. When the plasmonic–catalytic MIS junction exposures to the H_2_/air mixture with enough high concentration, the surface density of dipole charge becomes very large (up to ~10^12^ cm^−2^), and the generated electric field within the ultrathin oxide can be sufficiently strong so that appreciable electron tunneling does not occur. As illustrated in the top plot of Fig. 3b, the existence of dipole charges elevates the conduction energy band and gives rise to an increased Schottky barrier height. From the calculated electron profiles, it is observed that the density of the equilibrium electrons at the metal-oxide interface nearly equals to (~*n*_e_) that of the initial minority charge generated in metal, indicating most photoexcited electrons are trapped in the absorber rather than being ejected.

**Fig. 4:**
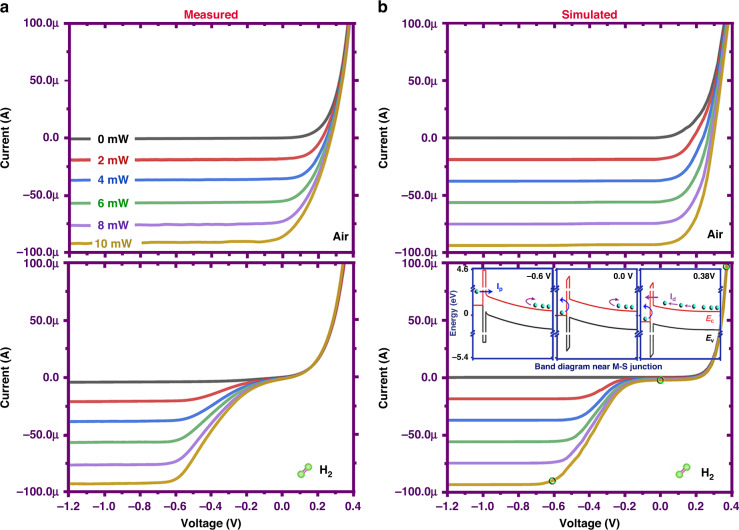
Quantitative description of the hydrogen-induced S-kink effect using quantum tunneling model. **a** Measured and **b** Simulated light *I*–*V* curves of the MIS junction operating in the inert air and hydrogen-containing mixture gas (3% H_2_ in air) with different illumination laser powers. The calculated energy band diagrams at three typical voltage biasing states are also present in the plot for the purpose of clarifying charge transfer mechanism for hydrogen-mediated junction response to external electrical biasing

A comprehensive comparison of the experimental and theoretical results of the dark/light *I*–*V* characteristics under air and H_2_ states is presented in Fig. 4a, b. From the measured light *I*–*V* curves, it was found that the scaling of the photocurrent verse light intensity is almost linear for both gas atmospheres. Based upon the above theoretical model, the simulation calculations are in good consistence with the experimental results. In particular, as demonstrated in the bottom plot of Fig. 4b, the kink effects at small voltages can be fully captured by introducing an additional dipole charge layer in the models, thus confirming the validity of the proposed mechanism on the hydrogen-mediated abnormal photoelectric behaviors. It should be also noted that, under different bias voltages, the impacts of hydrogen molecule on the light *I*–*V* curves are quite different. Under zero or positive bias voltages, the photocurrent response of the MIS junction observed in the hydrogen state is completely suppressed. In contrast, the photocurrent of the hydrogen state at a small reverse bias is only partially eliminated and it becomes comparable to that of the inert air state for large reverse bias voltages (*V*_b_ < −0.6 V). To understand these findings, the corresponding energy band diagrams for three typical biasing conditions were plotted in the insert. When applying a reverse external voltage to the MIS junction, the dipole-induced blocking field within the oxide layer can be canceled accordingly with the raise of the metal’s Fermi level, therefore leading to an increased tunneling possibility of hot electrons.

### Plasmonic–catalytic MIS junction used for hydrogen gas sensing

**Fig. 5:**
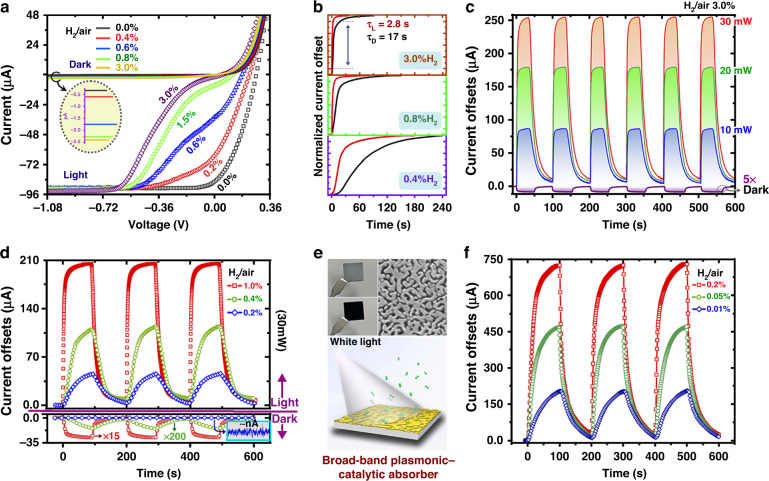
Hydrogen sensing performance of the plasmonic–catalytic MIS junction devices. **a** The stabilized light/dark *I*–*V* curves of the device under long-time exposure to gas atmospheres containing different hydrogen concentrations. **b** Time-dependent current response to hydrogen gases. The current offsets of the light and dark mode are normalized to their corresponding stabilized current values. The response rising time (refer to *τ*_L_ and *τ*_D_) is defined as the time for the incremental change in the output signal to go from 10% to 90% of its final value. **c,** **d** Dynamic response of the current for the device upon cyclic exposure to H_2_ and air purge under light and dark conditions. **e** Broad-band plasmonic–catalytic MIS junction hydrogen gas sensor based on Pt-Si nanoholes. (Top) Photographs and SEM image of the Pt/SiNH sample. **f** The sensing performance of the broad-band plasmonic devices for detecting low hydrogen content gases

Based upon the hydrogen-induced abnormal but interesting *I*–*V* kink effect, it is interesting to further explore the possibility of using the plasmonic–catalytic MIS junction as a unique and efficient photoelectric platform for hydrogen gas sensing. As shown in Fig. 5a, the light/dark *I*–*V* curves of the proposed sensor are characterized under gas atmospheres with different hydrogen concentrations. Note that, to acquire the stabilized electrical results, all the measurements on *I*–*V* curves were carried out after long enough reaction time. The dark *I*–*V* curves exhibit much smaller current offsets when varying the hydrogen concentration. Meanwhile, it is observed that the MIS junction operating in the dark can only response to a concentration larger than 0.4%, and tends to be saturated for a hydrogen concentration of 0.8%, indicating a poor hydrogen sensitivity and linearity. This is similar to the case of classic Schottky-based electrical hydrogen gas sensors. In such an electrical sensing scheme, it is usually necessary to elevate the temperature for achieving satisfied sensitivity and high response speed. In contrast, as clearly manifested by Fig. 5a, the combination of the plasmon excitation and catalytic MIS system leads to tens or even hundreds of times of enhancement on the current response. Increasing the hydrogen content in the mixture, the light *I*–*V* characteristic of the sensor evolves from that of a typical light-illuminated rectifying diode into a strong S-shaped property. The variation is more dramatical for small voltages, where the photocurrent decreases gradually with the increasing hydrogen concentration from 0% to 3%, revealing a large dynamic range for hydrogen gas detection. The involvement of hot electrons in the molecule–catalyst interaction process enables Pt to work as a photocatalyst in addition to its innate catalytic properties. Prior studies have proved that^45,50^, via charge transfer process, these energetic carriers can bring the adsorbate to vibrationally excited states and lower the activation barriers for dissociation or desorption. Apart from the enhanced sensitivity, fast response speed can also be guaranteed by the hot electron-induced photoelectric sensor. Note that, the time-dependent current characteristics of the light and dark conditions were measured by biasing the MIS junction device to 0 and −1 V, respectively. Considering the time-dependent current change as an indicator, it is believed that the hydrogen dissociation rates of the proposed sensor under plasmon excitation conditions are much higher than the dark cases at a given hydrogen exposure time. For a hydrogen concentration of 3%, the response time is found to be 2.8 s, which is around 6 times shorter than the dark condition as shown in Fig. 5b.

Figure 5c shows the dynamic current response for the device upon cyclic exposure to H_2_ (3%) and air purge under light and dark conditions. It was found that the response levels of the sensor for both light and dark operation modes are constantly maintained after several repeated cycles of exposure to hydrogen gas, suggesting its high reproducibility and repeatability (results for 30 min cyclic testing can be found in Supplementary Fig. S5). The current offset at each cyclic is around 2 μA for the dark operation mode, which is significantly smaller than that of the light illuminated cases. When increasing the laser power, the current offset increase accordingly in an almost linear manner. With 30 mW laser illumination, the enhancement factor of the current response over the dark case approaches 130. Straightforwardly, for detecting hydrogen at a very low concentration, the sensing performance can be improved by properly increasing the intensity of the incident light. In Fig. 5d, it is shown that the proposed plasmonic–catalytic MIS sensor illuminated with 30 mW monochromatic light is capable of detecting hydrogen gas with a concentration down to 0.2%. However, for the dark mode, i.e. the conventional electrical interrogation means, only a noise signal at ~nA level was observed at 0.2% concentration. The response enhancement from the plasmon excitation can be very significant especially for low concentration hydrogen content gas. For instance, at a hydrogen concentration of 0.4%, the current response of the light mode is around 1500 times larger than the dark mode.

It should be mentioned that the proof-of-concept device based on the grating structure is highly sensitive to wavelength, polarization and incident angle, which imposes a requirement for using high quality monochromatic laser source to meticulously match the excitation conditions of surface plasmon resonance. In previous studies^51^, broad-band plasmonic absorbers have been developed for near-infrared hot electron photodetection. As shown in Fig. 5e, the broad-band plasmonic structure consists of ultrathin metal-film that conformally formed on top of the random Si nanoholes (SiNHs). Compared to the elaborate periodic counterparts, such disordered nanostructures require relatively simple and low-cost fabrication process, and their optical properties are expected to be less sensitive to the incident angle and the polarization state. Most importantly, this hotspot-enriched disordered plasmonic nanosystem can be an ideal light-matter interaction platform to improve the surface dissociation rate for photocatalytic applications^52^. In these regards, the photoelectric sensing principle was extended into a more robust sensor design by incorporating the above broad-band plasmonic structures. The detailed fabrication process as well as the plasmonic property of metal/SiNHs can be found in previous publication^51^. In Fig. 5e, the appearance of the Pt/SiNHs based MIS sensor is almost completely black, indicating the excellent and broad-band absorption ability. The Pt/SiNHs based MIS junction device shares very similar *I*–*V* characteristics and hydrogen-induced kink effect as the grating structure. To evaluate its hydrogen sensing performance, a low-cost white LED lamp was adopted as the light source and the measurements on the cyclic response characteristics were performed under gas atmospheres with low hydrogen contents. The real-time current responses of the Pt/SiNHs-based MIS device during three cycles of gas injection are summarized in Fig. 5f. It was found that the proposed nanohole device operated at room temperature and zero-bias condition is capable to deliver repeatable and sensitive response to a very low hydrogen content of 0.01% that cannot be accurately and reliably detected by the grating device. Considering the factor of that, our device in standby state (hydrogen-free state) has a constant photocurrent output and its measurement accuracy determines the detect limit. Assuming a signal-to-noise ratio (SNR) of 1, the detection limit of the proposed nanohole device is found to 1.3 ppm with a response time of 10 s (refer to Supplementary Note 3 for more details). Overall, this alternative Pt/SiNH platform using white LED as light source possesses a number of advantages like low-cost, ease of design, high sensitivity, stable dynamic behavior, and especially without needs of electrical heating and external biasing (the performance comparison with other methods can be found in Supplementary Table 1). It may be further developed to use the environmental illumination if the background variation can be embedded in the characterization.

## Discussion

In this article, we report detailed studies of the interaction between chemical molecules and the plasmonic–catalytic MIS junction nanosystem with a focus on the hydrogen-tailored hot electron photoelectric behaviors. Upon plasmon excitation, the hydrogen injection on MIS junction leads to an abnormal light *I*–*V* characteristic that differs significantly from that of the measurements performed in inert air atmosphere. The S-shape kink observed in the *I*–*V* curves points to an inhibiting effect of hydrogen molecule on the hot electron collection process due to the formation of a dipole charge layer adjacent to the junction. A coupled optoelectrical simulation model was developed to unravel the underlying mechanisms, taking into account the interfacial dipole and quantum tunneling process at the MIS junction. It is concluded that the existence of interfacial dipole charges is the major determinant of the observed photoelectric kink effect, as it induces a strong blocking field that prevents the photogenerated hot electrons tunneling through the oxide. Our findings provide crucial insights into the complex mechanisms of interaction between molecules, plasmons and catalysts.

The conjunction of plasmonic hot carrier dynamics with catalytic reaction at an interfacial electrical structure not only offers fertile ground for fundamental science, but also holds great practical interest toward the development of more efficient photocatalysis and biochemical sensing strategies. Relying on the hydrogen-mediated kink effect, the plasmonic–catalytic MIS junction framework can be implemented as a robust photoelectric hydrogen gas sensor. Comparing to the conventional MIS or MS junction-based electrical gas sensor, this photoelectric sensing route possesses following advantages: (1) The involvement of hot electron dynamics in the metal catalyst can promotes the dissociation reaction of hydrogen molecule at surface, thereby allowing the device to obtain high response speed; (2) Based upon the hydrogen-tailored photoelectric conversion, the current offsets during cyclic testing can be as much as ~1500 times higher compared to that of the dark operation mode; (3) The proposed sensor device operated at the room temperature and zero-bias condition is capable to deliver repeatable and sensitive response to a very low hydrogen content at a ppm level, without needs of the electrical heating and external voltage biasing.

## Materials and methods

### Device fabrication

The nano-patterning process was performed on the single polished *n*-type (1–10 Ω‧cm) silicon wafer using Nikon NSR *i*-line stepper. The patterned photoresist (Shipley 1805, 500 nm thick) was used as the etch mask for reactive ion etching (RIE, Tegal 903e). After dry etching, standard RCA (SC1 and SC2) cleaning process was adopted to remove the damage, particles, and metal impurities of the patterned Si substrate. Meanwhile, RCA solutions can be considered as agents for wet chemical oxidation of the silicon due to the formation of ultra-thin silicon dioxide layer during above cleaning processes. Such a wet-chemically prepared oxide layer with a thickness around 1.2 nm is suitable to serve as the insulator for our MIS junction. Pt film with a target thickness of 50 nm and an additional thick electrode for wiring bonding were deposited on the structured silicon surface sequentially by magnetron sputtering and e-beam evaporation. To define the active area of the junction (~3 × 3 mm^2^), two shadow masks with square and U-shape apertures were used in above physical deposition processes. Aluminum film (300 nm) was deposited on the rear side of wafer, which serves as the Ohmic contact (cathode) for the *n*-type silicon. The wafers were then cut into small pieces (1.5 cm × 1.5 cm) using the dicing saw. The complete chips were mounted on printed circuit board (PCB) and the devices were linked electrically with the PCB pins by wire-bonding. The SiNH-based devices were fabricated in almost the same procedure as the grating-based device except for the silicon nanostructure, which is made with our previously developed method^51^.

### Optical simulation

The reflection spectra and the absorption power density distribution were simulated by using the COMSOL Multiphysics software package. The structure parameters are given in the figure caption of Fig. 1. The angle-dependent reflection spectra and the theoretical dispersion relation of the plasmonic resonance are discussed and compared to the experimental results in Supplementary Note 1 (Fig. S1). The simulated spectra of light absorption in Pt and silicon are discussed together with the EQE (see Supplementary Note 2 and Fig. S2 for more details).

### Optical and optoelectrical measurements

The experimental setup for the hydrogen sensing is depicted in Fig. S3. The flow rates of air gas and hydrogen-containing gas are controlled by two high accuracy gas flow meters. The final mixture gas and air gas are fixed to 100 sccm in the experiments. The MIS junction sample is mounted inside a home-made gas chamber with the diameter of 5 cm and thick of 0.4 cm (inner dimension). NKT super continuum laser combined with AOTF was used to generate monochromatic light. To obtain the zero-order reflection, the tunable laser beam strikes grating sample and reflects into an optical power meter to measure of the intensity (specular zero-order reflection). The gas chamber was equipped with feed-throughs for electrical connections to the source meter (Keithley 2636B, Keithley Instruments, Inc.), allowing for the real-time tracking of the current changes.

### Quantum tunneling model of the MIS junction

Coupled electrical and optoelectrical simulations were performed based upon the drift-diffusion transport framework using commercial TCAD tools (Silvaco ATLAS). A degenerate semiconductor with near-zero band gap and high electron affinity was adopted to qualitatively represent the metallic Pt absorber. The tunneling processes is included in the insulating layer (1 nm SiO_2_ by natural oxide growth), where a non-local quantum barrier tunneling model was applied to the MIS junction area in simulations. The electron affinity of semiconductor absorber and the non-local tunneling mass are considered as the main fitting parameters for correlating the simulated dark *I*–*V* curve from experimental one. The plasmon-induced hot carriers are modeled as the photogenerated electron–hole pairs in above metallic-like semiconductor absorber. The absorber is assumed to be a *p*-type doped semiconductor. For simplicity, constant optical generation rates (*G*_opt_, cm^−3^ s^−1^) have been assigned to the whole metallic absorber. In accordance to the incident laser power (*P*_0_) used in experiments, the input values of *G*_opt_ is defined as *P*_0_*A*_t_*η*_eqe_/*E*_p_*V*_a_, where *E*_p_ is photon energy at 1064 nm, *A*_t_ is the optical absorption deduced from Fig. S1, *V*_a_ is the active illuminated volume of absorber, and *η*_eqe_ is equivalent to previously obtained quantum efficiency, which can be regarded as a correction factor for taking into account the relative less efficient processes of hot electron transfer. The most critical physical parameters used in our quantum simulations are provided in Supplementary Table S2.

## Supplementary information

Supplementary Information

## References

[1] Yue ML (2021). Hydrogen energy systems: a critical review of technologies, applications, trends and challenges. Renew. Sustain. Energy Rev..

[2] Majumdar A (2021). A framework for a hydrogen economy. Joule.

[3] Liu WG (2021). The production and application of hydrogen in steel industry. Int. J. Hydrog. Energy.

[4] Van Hoecke L (2021). Challenges in the use of hydrogen for maritime applications. Energy Environ. Sci..

[5] Pearton SJ, Ren R (2012). Gallium nitride-based gas, chemical and biomedical sensors. IEEE Instrum. Meas. Mag..

[6] Hübert T (2011). Hydrogen sensors–a review. Sens. Actuators B Chem..

[7] Buttner WJ (2011). An overview of hydrogen safety sensors and requirements. Int. J. Hydrog. Energy.

[8] Nugroho FAA (2019). Metal–polymer hybrid nanomaterials for plasmonic ultrafast hydrogen detection. Nat. Mater..

[9] Messmer RP (1977). The interaction of atomic hydrogen with Ni, Pd, and Pt clusters. Chem. Phys. Lett..

[10] Baldi A (2014). In situ detection of hydrogen-induced phase transitions in individual palladium nanocrystals. Nat. Mater..

[11] Duan XY, Kamin S, Liu N (2017). Dynamic plasmonic colour display. Nat. Commun..

[12] Bosko ML (2021). Advances in hydrogen selective membranes based on palladium ternary alloys. Int. J. Hydrog. Energy.

[13] Gu HS, Wang Z, Hu YM (2012). Hydrogen gas sensors based on semiconductor oxide nanostructures. Sensors.

[14] Koo WT (2020). Chemiresistive hydrogen sensors: fundamentals, recent advances, and challenges. ACS Nano.

[15] Luo YF (2017). Hydrogen sensors based on noble metal doped metal-oxide semiconductor: a review. Int. J. Hydrog. Energy.

[16] Ivanov II (2021). Investigation of catalytic hydrogen sensors with platinum group catalysts. Sens. Actuators B: Chem..

[17] Beni T (2019). Metamaterial for hydrogen sensing. ACS Sens..

[18] Simon I, Arndt M (2002). Thermal and gas-sensing properties of a micromachined thermal conductivity sensor for the detection of hydrogen in automotive applications. Sens. Actuators A Phys..

[19] Korotcenkov G, Han SD, Stetter JR (2009). Review of electrochemical hydrogen sensors. Chem. Rev..

[20] Potje-Kamloth K (2008). Semiconductor junction gas sensors. Chem. Rev..

[21] Wang GP, Dai JX, Yang MH (2021). Fiber-optic hydrogen sensors: a review. IEEE Sens. J..

[22] Liang TT (2022). Highly sensitive hydrogen sensing based on tunable diode laser absorption spectroscopy with a 2.1 μm diode laser. Chemosensors.

[23] Brolo AG (2012). Plasmonics for future biosensors. Nat. Photon..

[24] Cetin AE (2014). Handheld high-throughput plasmonic biosensor using computational on-chip imaging. Light Sci. Appl..

[25] Hu X (2016). Metamaterial absorber integrated microfluidic terahertz sensors. Laser Photon. Rev..

[26] Lopez GA (2017). Recent advances in nanoplasmonic biosensors: applications and lab-on-a-chip integration. Nanophotonics.

[27] Liang L (2018). Unity integration of grating slot waveguide and microfluid for terahertz sensing. Laser Photon. Rev..

[28] You EM (2021). Nanobridged rhombic antennas supporting both dipolar and high-order plasmonic modes with spatially superimposed hotspots in the mid-infrared. Opto-Electron. Adv..

[29] Liu N (2011). Nanoantenna-enhanced gas sensing in a single tailored nanofocus. Nat. Mater..

[30] Etezadi D (2017). Nanoplasmonic mid-infrared biosensor for in vitro protein secondary structure detection. Light Sci. Appl..

[31] Matuschek M (2018). Chiral plasmonic hydrogen sensors. Small.

[32] Kazuma E (2018). Real-space and real-time observation of a plasmon-induced chemical reaction of a single molecule. Science.

[33] Liang L (2021). A programmable DNA-silicification-based nanocavity for single-molecule plasmonic sensing. Adv. Mater..

[34] Long SQ (2022). Sensing absorptive fluids with backside illuminated grating coupled SPR sensor fabricated by nanoimprint technology. Sens. Actuators A Phys..

[35] Chan GH (2007). Plasmonic properties of copper nanoparticles fabricated by nanosphere lithography. Nano Lett..

[36] Kim JM (2021). Synthesis, assembly, optical properties, and sensing applications of plasmonic gap nanostructures. Adv. Mater..

[37] Torrini F (2022). Imprinted biopolymers as green abiotic route in immunoglobulin affinity plasmonic sensing. Biosens. Bioelectron..

[38] Tittl A (2018). Imaging-based molecular barcoding with pixelated dielectric metasurfaces. Science.

[39] Yesilkoy F (2019). Ultrasensitive hyperspectral imaging and biodetection enabled by dielectric metasurfaces. Nat. Photon..

[40] Wen L (2019). Multiband and ultrahigh figure-of-merit nanoplasmonic sensing with direct electrical readout in Au-Si nanojunctions. ACS Nano.

[41] Brongersma ML, Halas NJ, Nordlander P (2015). Plasmon-induced hot carrier science and technology. Nat. Nanotechnol..

[42] Wen L (2017). Enhanced photoelectric and photothermal responses on silicon platform by plasmonic absorber and omni-schottky junction. Laser Photon. Rev..

[43] Wang P (2018). Reactive tunnel junctions in electrically driven plasmonic nanorod metamaterials. Nat. Nanotechnol..

[44] Zhang XM (2013). Plasmonic photocatalysis. Rep. Prog. Phys..

[45] Zhang YC (2018). Surface-plasmon-driven hot electron photochemistry. Chem. Rev..

[46] Chen Q (2020). On-chip readout plasmonic mid-IR gas sensor. Opto-Electron. Adv..

[47] Weidemann O (2003). Influence of surface oxides on hydrogen-sensitive Pd: GaN Schottky diodes. Appl. Phys. Lett..

[48] Gaman VI (2008). Basic physics of semiconductor hydrogen sensors. Russian Phys. J..

[49] Irokawa Y (2010). Hydrogen-induced change in the electrical properties of metal-insulator-semiconductor Pt-GaN diodes. J. Appl. Phys..

[50] Mukherjee S (2013). Hot electrons do the impossible: plasmon-induced dissociation of H_2_ on Au. Nano Lett..

[51] Wen L (2018). Hot electron harvesting via photoelectric ejection and photothermal heat relaxation in hotspots-enriched plasmonic/photonic disordered nanocomposites. ACS Photon..

[52] Jiang XY (2022). Plasmonic active “hot spots”-confined photocatalytic CO_2_ reduction with high selectivity for CH_4_ production. Adv. Mater..

