# Supplementary material for: On-chip ultrasensitive and rapid hydrogen sensing based on plasmon-induced hot electron–molecule interaction
Source: Light Sci Appl. 2023 Mar 21;12:76. doi: 10.1038/s41377-023-01123-4 (PMC10030554; doi:10.1038/s41377-023-01123-4)
Supplement: Supplementary file 1 — Supplementary Information [file 41377_2023_1123_MOESM1_ESM.docx]

Supplementary Information for

**On-Chip Ultrasensitive and Rapid Hydrogen Sensing Based on**

**Plasmon-Induced Hot Electron-Molecule Interaction**

Long Wen**^1^**, Zhiwei Sun**^1^**, Qilin Zheng**^1^**, Xianghong Nan**^1^**, Zaizhu Lou**^1^**,

Zhong Liu**^2^**, David R. S. Cumming**^3^** , Baojun Li**^1^** and Qin Chen**^1*^**

**^1^**Guangdong Provincial Key Laboratory of Nanophotonic Manipulation, Institute of Nanophotonics, Jinan University, Guangzhou 511443, China

**^2^**College of Life Science and Technology, Jinan University, Guangzhou 510632, China

**^3^**School of Engineering, University of Glasgow, Glasgow G12 8QQ, UK

**^*^**Correspondence: [chenqin2018@jnu.edu.cn](mailto:chenqin2018@jnu.edu.cn)

**Table of Contents:**

**Supplementary Note 1** Grating-coupled surface plasmon resonance

**Supplementary Note 2** Silicon photovoltaic response

**Supplementary Note 3** Detection limit of sensor

**Supplementary Figure S1** Optical properties at oblique angles of incidence

**Supplementary Figure S2** An evaluation of the contribution of Silicon response

**Supplementary Figure S3** Experimental setup for the optoelectric hydrogen sensing

**Supplementary Figure S4** DL analysis of the plasmonic-catalytic sensors

**Supplementary Figure S5** Long-time cyclic detection of hydrogen gas

**Supplementary Table S1** Performance comparison on different methods

**Supplementary Table S2** Electrical parameters used in the TCAD simulations

**Supplementary Note 1** Grating-coupled surface plasmon resonance

The plasmonic resonance of the periodically corrugated metallic surface structure coupled by grating diffractive order is well described by momentum matching conditions:

$k\sin\theta+mG=\pm k_{\mathrm{sp}}$ (1)

where *θ* is incident angles, *G* is grating constant ($2\pi/P$), and *k* is the wave vector ($2\pi n/\lambda$, n is the refraction index of incident medium). As the ultra-shallow surface relief (H<*λ*/10) can be considered as small perturbations to the flat metal, the wave vector *k*_sp_ therefore can be approximately calculated using the dispersion relationship of the plasmonic mode supported by flat metal/dielectric interface:

$k_{\mathrm{sp}}=k\sqrt{\varepsilon_{D}\varepsilon_{M}/(\varepsilon_{D}+\varepsilon_{M})}$ (2)

where *ε*_M_ is the metal relative permittivity and *ε*_D_ is the relative permittivity of the surrounding dielectric medium (air) around the Pt.


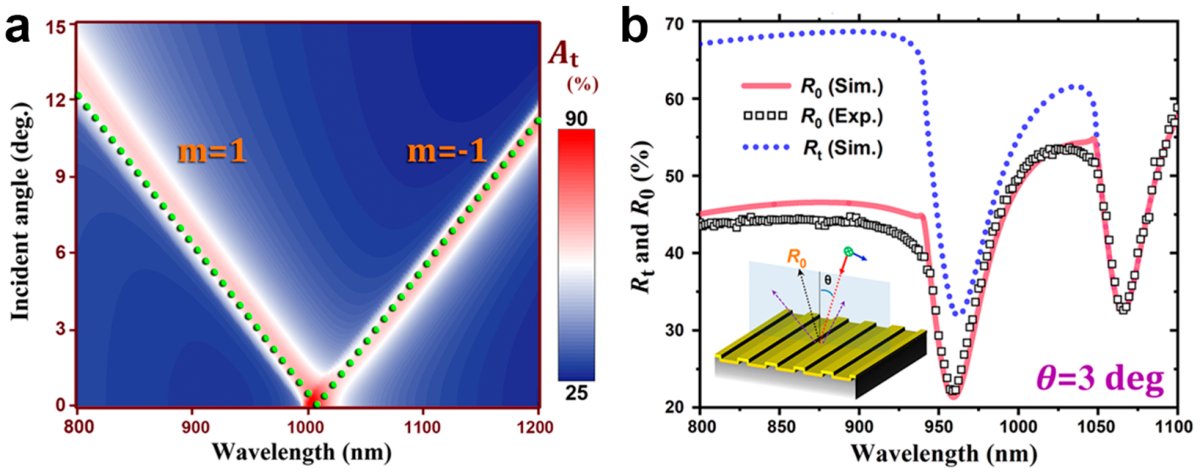


**Supplementary Fig. S1 Optical properties of the grating-based plasmonic structure at oblique angles of incidence.** **a** Numerical absorption spectra as functions of wavelength and incident angle. The green dotted lines superposed on the contour plots represent the predictions from plasmonic coupling formulas. **b** Experimental and numerically fitted zero-order reflection curves (*R*_0_) at oblique incident angle (*θ*=3 degrees).

The resulting resonance wavelength versus incident angle predicted by above formulas is superimposed over the simulated absorption spectra in **Fig. S1a**, where theoretically predicted resonances are closely matched with the simulated maximum absorption positions. As shown in **Fig. S1b**, we also performed the optical measurements on the zero-order reflectance of the plasmonic structures under oblique incident angle, which shows a good match with the simulated result (fitting parameters are summarized in the main text). For the oblique incidence, the plasmonic resonance will split into two strong absorption peaks that are attributed to the coupling with the positive and negative diffraction orders.

**Supplementary Note 2.** Silicon photovoltaic response

Based upon the fitted geometric parameter of the proposed plasmonic device, our optical simulation indicates the absorption takes place mainly in the lossy Pt layer. As shown in **Fig. S2**a, while the total absorption at 1064 nm is found to be ~70%, the contribution from silicon absorption is ultra-small as the transmission portion is only ~2%. To provide a quantitative assessment of the silicon response, we also carried out a coupled opto-electrical simulation based on TCAD models (Silvaco Atlas). The detailed model descriptions and parameters about the junction can be found in the “Materials and methods” sections of main text. The substrate thickness is assumed to be 350 μm. The light transmits into silicon and follows the Beer’s absorption law, which was modeled using ray tracing method. The optoelectric modeling was carried out in wavelength domain as shown in **Fig. S2**b. The light transmission obtained from optical simulation was used as the input source, no surface reflection needed to be further considered in the ray tracing model. The resulting EQE at 1064 nm is only ~0.02%, which corresponds to a photocurrent responsivity of 0.19 mA W^-1^. The low EQE observed at 1064 nm is due to both the insufficient light absorption and the internal losses associated to large absorption depth (much larger than the effective diffusion length of minority carriers in the *n*-type Si substrate).


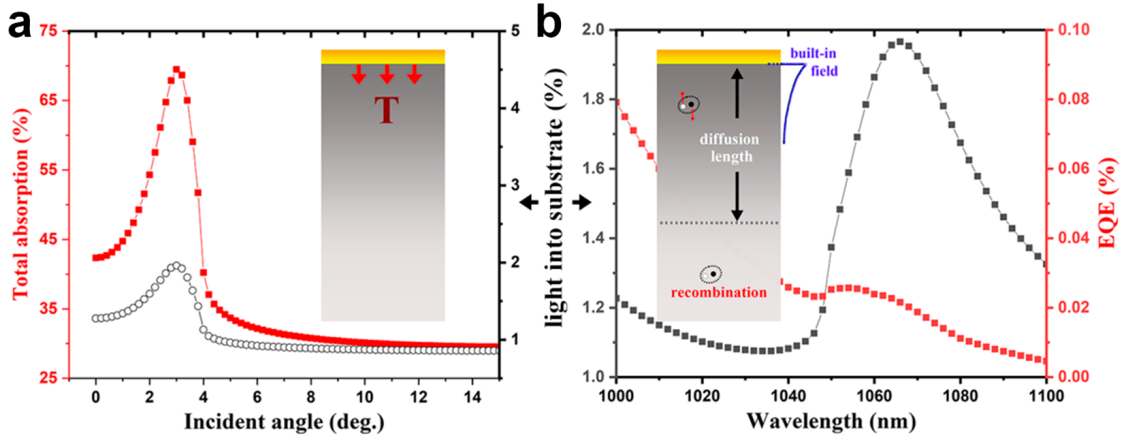


**Supplementary Fig. S2 An evaluation of the contribution of Silicon response.** **a** The calculated angle-dependent total absorption of plasmonic structure and the light transmitted into the silicon substrate. **b** Calculated EQE and silicon absorption as a function of wavelength for the catalytic-plasmonic MIS junction device (*θ*=3 degrees).


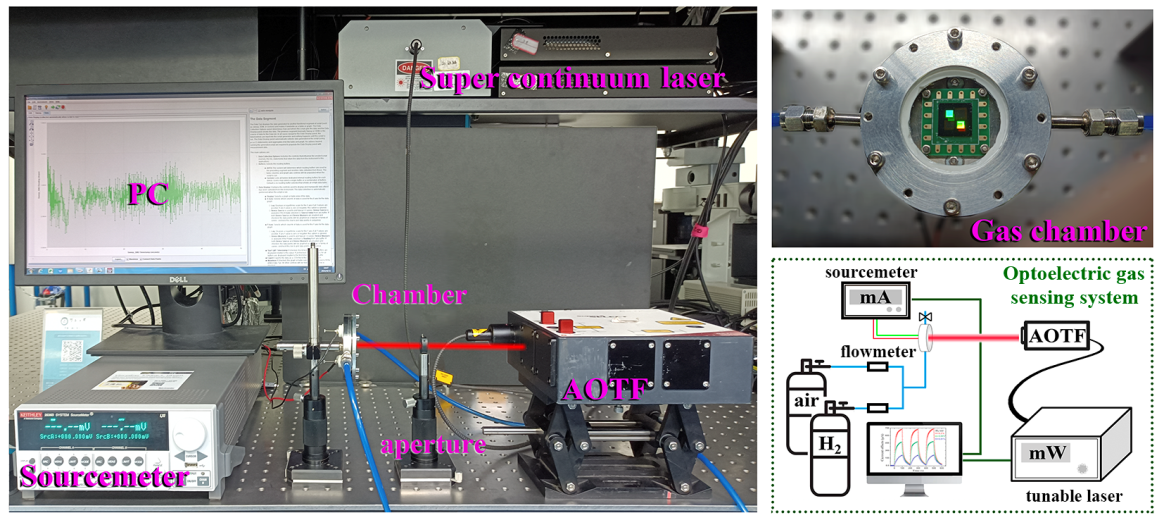


**Supplementary Fig. S3** Experimental setup for the optoelectric hydrogen sensing. Top right of the figure shows the plasmonic sensor mounted in a home-made gas chamber.

**Supplementary Note 3** Detection limit

The plasmonic-catalytic hydrogen sensor with random hotspots-enriched structures has an improved sensitivity and speed as demonstrated in the main text. In order to evaluate the possibilities of applying such a novel photoelectric type hydrogen sensor for ~ppm level detection, further analysis on the possible detection limit were performed. The temporal responses of the device under different hydrogen gas concentrations (from 100 ppm to 0.6%) are summarized in **Fig. S4a**. As shown in the plots, the device reaches the saturation state (*i.e.*, completely suppressed photocurrent response) quickly for the concentration of 0.6 %, and is capable to yield a current offset with tens to hundreds of micro-amps within only 10 seconds. Based on these results, in **Fig. S4c**, we plot the hydrogen-induced current offsets for different gas concentrations assuming a sensing time of 10 s. Note that, due to limited accuracy of our gas flow meters, the responses for ultra-low level (*e.g.* 1 ppm) are extrapolated from the available experimental results. The predicted current offset of the sensor in detection of 1 ppm concentration within 10 s is found to be 0.18 μA. Considering the factor of that, our device in standby state (hydrogen-free state) has a constant photocurrent output and its measurement accuracy determinates the detect limit of our sensor. At a sampling duration of 100 s, the photocurrent output shows a standard deviation (δ) of 0.25 μA (**Fig. S4b**). The detection limit (signal-to-noise ratio of 1, Δ*I*=δ) of the proposed sensor is around 1.3 ppm.


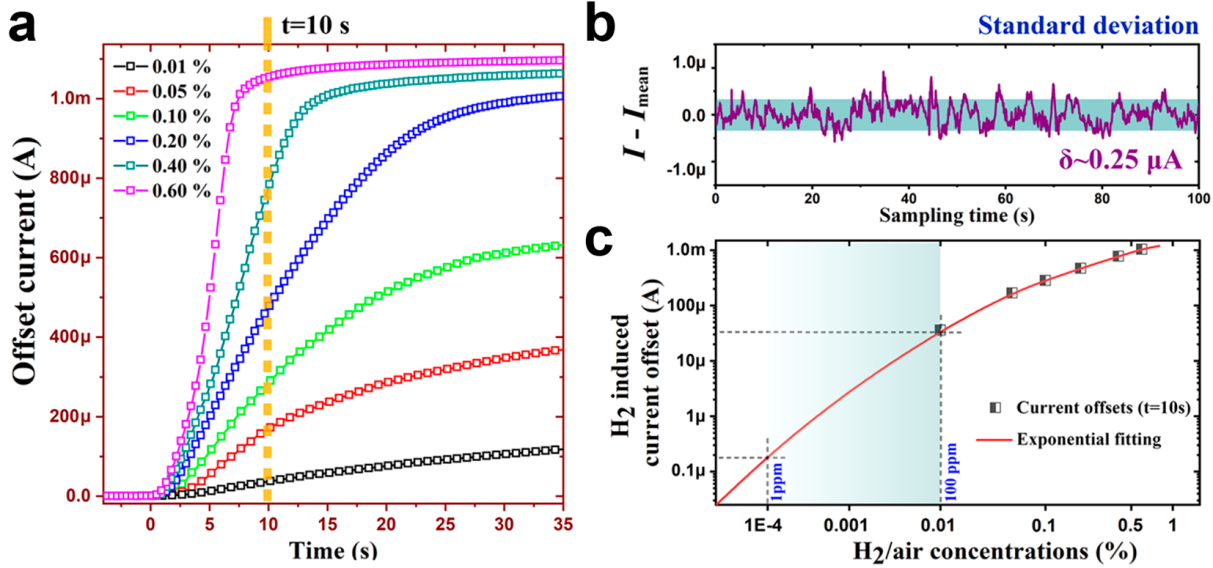


**Supplementary Fig. S4 DL analysis of the plasmonic-catalytic sensors.** **a** Time-dependent current response to hydrogen gases with different concentrations. **b** Photocurrent variation as a function of time for the device operated at hydrogen-free condition. **c** Predicted current offsets at hydrogen gas at ~ppm levels.


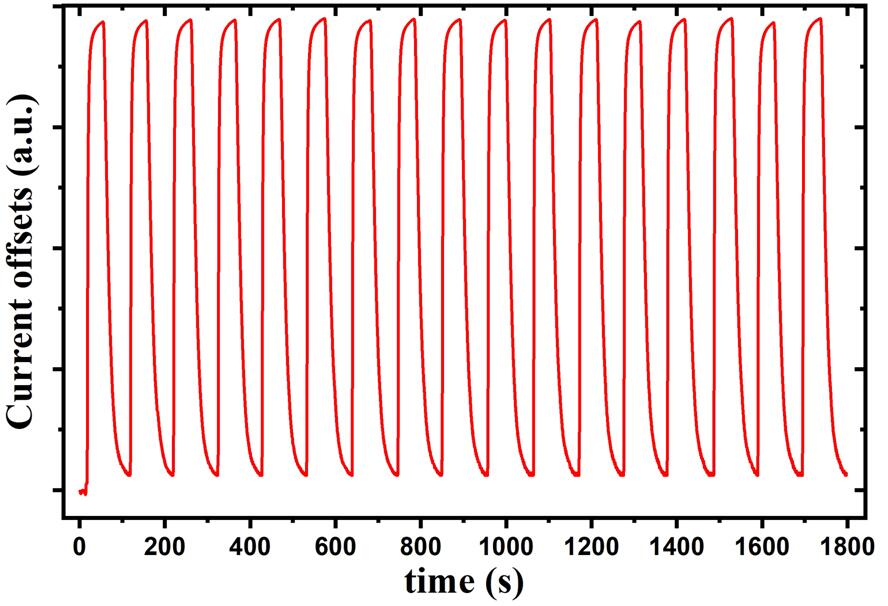


**Supplementary Fig. S5 Cyclic detection of hydrogen gas in 30 minutes.** Response/recovery time of each cycle is set to 100 s. (1064 nm laser power: 30 mW; H_2_ gas concentration: 3%).

**Supplementary Table S1** Performance comparison on different methods


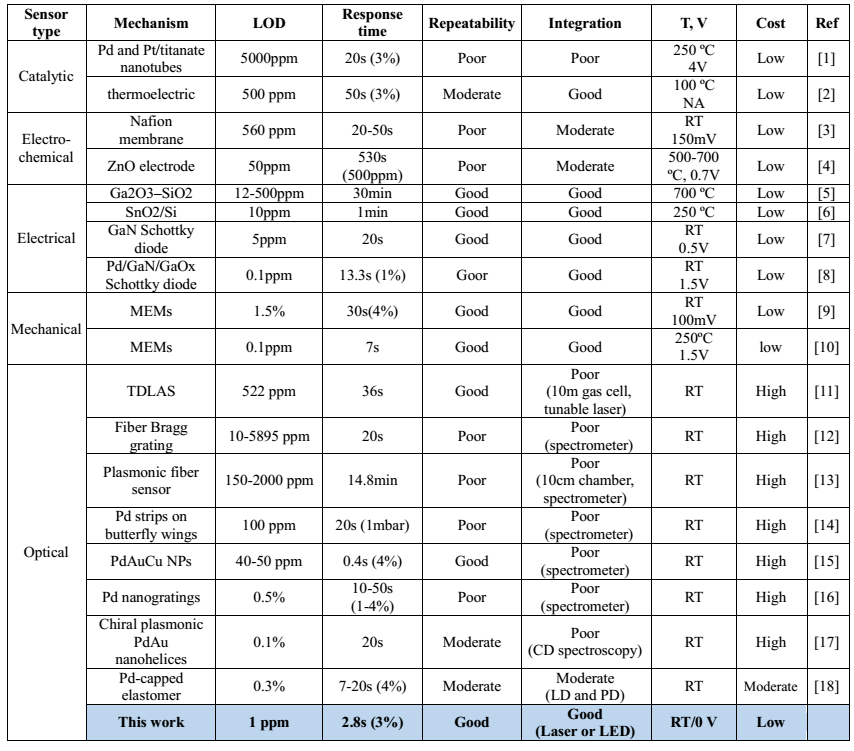


**Supplementary Table S2** Electrical parameters used in TCAD simulation

| **Bulk material properties:** | | | | |
| --- | --- | --- | --- | --- |
|  | Metal | Oxide | Silicon | Description |
| *N*_d_ | 1×10^21^ | / | 5×10^15^ | Doping level, cm^-3^ |
| *ε* | 11.8 | 3.9 | 11.8 | Relative epsilon |
| *χ* | 4.87 | 0.9 | 4.17 | Electron affinity, eV |
| *E*_g_ | 0.001 | 9.0 | 1.08 | band gap, eV |
| *N*_c_ | 6×10^5^ | / | 2.8×10^19^ | Conduction/valence band  density of states, cm^-3^ |
| *N*_v_ | 1.04×10^21^ | / | 1.04×10^19^ |  |
| *m*_e_ | / | 0.1 m_0_ | / | Non-local tunneling mass |
| *m*_h_ | / | 0.1 m_0_ | / |  |
| *τ*_e_ | 1×10^-9^ | / | 1×10^-7^ | Minority carrier lifetimes, s |
| *τ*_h_ | 1×10^-30^ | / | 1×10^-7^ |  |
| *μ*_e_ | 1.00 | / | 1000 | Carrier mobility, cm^2^ v^-1^ s^-1^ |
| *μ*_h_ | 0.01 | / | 500 |  |
| **Interface/surface properties:** | | | | |
| Metal/Oxide interface | | +*Q*_s_/-*Q*_s_, *Q*_s_=6×10^12^ cm^-2^ | | Hydrogen-induced dipole |
| Silicon surface layer | | *τ*_SRH_=2×10^-9^ s | | Shockley-Read-Hall lifetime |
